# *Lomentospora prolificans* endocarditis - case report and literature review

**DOI:** 10.1186/s12879-016-1372-y

**Published:** 2016-01-29

**Authors:** Melissa Kelly, Robert Stevens, Pamela Konecny

**Affiliations:** 1 Department of Infectious Diseases and Immunology, St George Hospital, Kogarah, NSW 2217 Australia; 2 Microbiology Department, South Eastern Area Sydney Laboratories, St George Hospital, Kogarah, NSW 2217 Australia; 3 Department of Infectious Diseases and Immunology & St George Clinical School, Faculty of Medicine, University of New South Wales, St George Hospital, Kogarah, NSW 2217 Australia

**Keywords:** Fungal endocarditis, *Lomentospora prolificans*, *Scedosporium prolificans*, Voriconazole, FDG-PET scan, Invasive fungal disease, Oncology

## Abstract

**Background:**

*Lomentospora prolificans* (formally *Scedosporium prolificans)* is an environmental mould with a global distribution. Endocarditis caused by *L. prolificans* is a rare but serious emerging disease in immunocompromised patients. Prior to this case there have only been eight cases reported in the literature. Diagnosis can be challenging and there are no evidence-based guidelines for treatment.

**Case presentation:**

We report a 75-year-old woman with ovarian carcinoma who presented with fever after chemotherapy. Repeated sterile site cultures remained negative until day 22 of admission, when *Lomentospora prolificans* was isolated from blood cultures. Following extensive investigations, including Fluoro-D-glucose positron emission tomography (FDG-PET) and transoephageal echocardiography (TOE), the patient was diagnosed with endocarditis complicated by cerebral emboli. The patient was considered unsuitable for surgical intervention and passed away five days after the fungus was isolated.

**Conclusion:**

Endocarditis caused by *Lomentospora prolificans* is a rare but emerging condition, with limited treatment options and a high mortality. Awareness of the increasing incidence of *Lomentospora prolificans* infection, diagnosed often at an advanced stage, with potential for endocarditis may prompt earlier echocardiography or FDG-PET imaging. Further studies are needed to determine the optimal combination and duration of anti-fungal agents, used in conjunction with aggressive surgical excision where feasible.

**Electronic supplementary material:**

The online version of this article (doi:10.1186/s12879-016-1372-y) contains supplementary material, which is available to authorized users.

## Background

*Lomentospora prolificans,* formally referred to as *Scedosporium prolificans*, is a dermatiaceous mould with a global distribution, and is an emerging pathogen linked to severe infections, particularly in the immunocompromised [1, 2]. Recently in Australia, *L. prolificans* was reported amongst the most frequently isolated invasive mould infections, possibly related to the abundance in soil, particularly in urban environments [3]. Traditional risk factors for infection include lung disease, malignancy, transplantation, trauma and HIV positivity [1–3]. More recently, patients with chronic lung disease and rheumatological conditions may be considered at-risk [3]. Epidemiological data is emerging to include exposure to antifungal prophylaxis, admission to the intensive care department and proximity to hospital construction work as likely predisposing factors for infection [3].

Recent reclassification of *Scedosporium prolificans* to *Lomentospora prolificans* arose from fundamental changes in the International Code of Nomenclature based on phylogenetic profiling and combined international mycology working party recommendations in 2014 [4]. *L. prolificans* is distinguished from the other *Scedosporium* species both by morphological and clinical features. Microscopically, the annelids have a typically swollen or inflated rather than tubular base with apical flask-shaped conidia and macroscopically a different colony texture and colouration. Clinically, *Lomentospora* cause serious disseminated infections, particularly in immunosuppressed hosts. Its typical high-level in-vivo resistance to voriconazole sets it apart [4].

Clinical manifestations are varied. Focal bone and joint infections in post-traumatic or post-operative patient groups have been well described in the immunocompetent host. Disseminated disease is more common in the immunosuppressed cohort and can present in myriad forms. Respiratory, ophthalmic, cerebral, skin and soft tissue lesions along with endocarditis have been described [1]. Generally, *Scedosporium* spp are readily isolated from sterile site culture and identified by their characteristic macroscopic and microscopic morphology. Despite this, blood culture isolation is typically late in the illness and thus has limited diangnostic utility, though blood culture positivity may indicate widely disseminated disease [2]. Infective endocarditis caused by *L. prolificans* has rarely been identified [1, 3, 5].

Diagnosis of endocarditis depends upon clinical criteria, microbiological sampling and imaging, typically with transoesophageal echocardiography (TOE). The role of Fluoro-D-glucose positron emission tomography (FDG-PET) in the diagnostic armamentarium for fever of unknown origin, including endocarditis is emerging [6]. Recently, the European Society of Cardiology Guidelines have incorporated FDG-PET scanning in the diagnostic algorithm for prosthetic valve endocarditis when the diagnosis is unable to be reached definitively with traditional criteria [7]. Furthermore, high sensitivity and good specificity, allow non-invasive FDG-PET scanning to guide more targeted invasive diagnostics such as transoesophageal echocardiography, as in this case.

## Case presentation

We report a 75-year-old female with stage IV poorly differentiated ovarian carcinoma diagnosed in 2000, on palliative carboplatin-based chemotherapy since 2011. Past treatment included bilateral salpingo-oopherectomy and hysterectomy, ileostomy due to small bowel obstruction with subsequent fistulae formation and bilateral nephrostomy drainage after radiation-induced ureteric obstruction. She had experienced recurrent urinary tract infections and nephrostomies were previously colonised with methicillin resistant *Staphylococcus aureus* (MRSA), *Staphylococcus epidermidis & Candida albicans.* She had never received anti-fungal prophylaxis. Co-morbidities included hypertension, multiple pulmonary emboli on enoxaparin and paroxysmal atrial fibrillation. There were building works underway in the hospital during her hospital admission, although she was not in the immediate vicinity.

The patient was admitted to hospital due to a brief syncopal episode with associated lethargy, nausea, light-headedness and increased watery ileostomal output following chemotherapy one week earlier. Clinical examination including central nervous system was unremarkable, and she was haemodynamically stable and afebrile. Laboratory investigations revealed sodium 127 mg/mL (135–145 mg/mL), creatinine 145 mg/mL (45–90 mg/mL), haemoglobin 87 g/L (115–165 g/L), white cell count 4.52 cells/μl (3.9–11.1 cells/μl) and lymphocyte count 0.7 cells/μl (1.0–4.0 cells/μl). On day 2 of admission, she developed fevers of 38.5 °C and was empirically commenced on piperacillin-tazobactam and vancomycin. *Staphylococcus epidermidis* isolated from the nephrostomy fluid was considered colonisation and the nephrostomies were changed. Initial septic screen was non-contributory, including negative faecal microscopy.

**Fig. 1:**
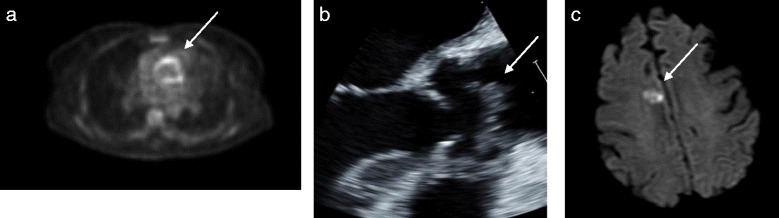
Diagnostic imaging of *Lomentospora prolificans* endocarditis. **a** FDG-PET axial image showing abnormal linear uptake in the aortic root and the ascending aorta and **b** Transoesophageal echocardiogram (still shot) axial view demonstrating a large pedunculated mass within the ascending aorta arising from the commissure of the right and non-coronary cusp of the aortic valve with a calcified base; and cerebral emboli **c** MRI brain axial view, with some motion artefact, showing post-gadolinium enhancement of the right parasagittal frontal lobe consistent with an embolic lesion

Persisting fever during her third week of admission, with negative sterile site cultures 14 days into her admission, prompted assessment for metastatic disease progression with FDG-PET scan. There was no evidence of metastatic malignant disease detected, but an area of abnormal avidity was identified on the aortic valve (Fig. 1a). Subsequent transthoracic echocardiogram (TTE) revealed a small mobile echo-density superior to the aortic valve. Transoesophageal echocardiogram (TOE) (Fig. 1b) revealed a pedunculated mass within the ascending aorta arising from the commissure of the right & non-coronary cusp of the aortic valve with a calcified base (20 × 14 × 11mm). An additional movie file shows this in more detail [see Additional file 1].

The patient became delirious on day 17 with a fluctuating level of consciousness. Brain MRI identified a contrast-enhancing right parasagittal frontal lobe lesion consistent with an embolic lesion (Fig. 1c).

**Fig. 2:**
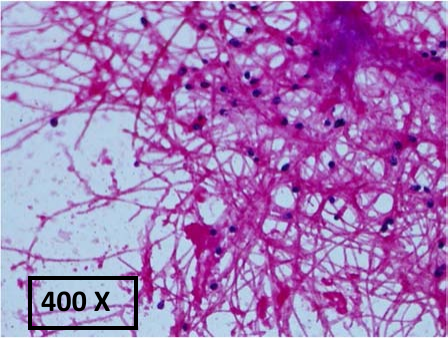
Direct Gram stain from the aerobic blood culture bottle at 72 h showing hyphae, conidiophores and conidia consistent with *Lomentospora prolificans* (400 × magnification)

On day 22 of admission, fungi were isolated from repeat blood cultures after 3 days of incubation using the automated BacT/ALERT 3D system (bioMerieux, USA) subsequently morphologically identified as *Lomentospora prolificans* (Fig. 2). Identification was based on classical morphological findings from colonies grown on potato dextrose agar. The fungus had high minimum inhibitory concentration (MIC) to amphotericin (>32 mg/l) and itraconazole (>32 mg/L), and in-vitro sensitivity to voriconazole (0.5 mg/L) using Etest methodology (bioMerieux, USA).

The patient was commenced on therapy with voriconazole (loading dose 600 mg IV 12 hourly). Unfortunately, due to her underlying advanced malignancy, disseminated fungal infection and poor clinical status, she was considered unsuitable for surgical management. She was managed with palliative intent and passed away five days after the fungus was isolated on day 27 of her admission.

## Discussion

Endocarditis caused by *Lomentospora prolificans* is rare. A Medline search was conducted using the terms *Scedosporium* invasive infection, *Scedosporium* endocarditis, *Scedosporium prolificans* endocarditis and disseminated *Scedosporium* and *Lomentospora* endocarditis and infection. Eight cases were identified [5, 8–10]. Three patients had underlying cardiac pathology: a prosthetic aortic valve, an implanted permanent pacemaker or a history of rheumatic disease. Four patients were immunosuppressed. Of these, three had haematological malignancies and one had previously received a renal transplant. One patient was an injecting drug user. There were four men and four women, with a mean age of 42 (range 29–75 years). The majority of patients presented with fever, embolic phenomenon and vegetations which were readily identified on echocardiography. Reported complications included septic arthritis, endophthalmitis and meningitis, along with cerebral, splenic and pulmonary emboli. All except for two cases were treated with a combination of liposomal amphotericin and either a triazole or flucytosine. Two patients were treated with voriconazole as the primary antifungal agent. Surgical intervention was undertaken in half the cases. Of those, only the patient whose infected pacemaker was removed survived. Presentation is typically at an advanced stage of dissemination and diagnostically challenging. Mortality was high at 75 % (6/8) and immunocompetence appeared to increase survival [5, 8–10]. The table below includes our case (Table 1).

**Table 1:** Cases of *Lomentospora (Scedosporium) prolificans* endocarditis 1990 – 2014

Case	Year	Age/Gender	Predisposition	Valve	Complications	Treatment/Surgery	Outcome
1^(5)^	1990	30 male	Intravenous drug user	Mitral	Septic arthritis	AmBisome	Survived
						Flucytocine	
2^(5)^	1997	67 female	Prosthetic aortic valve	Aortic	Cerebral emboli	AmBisome	Died
						Fluconazole	
3^(5)^	2001	52 female	Multiple myeloma	Aortic	Endophthalmitis, intracranial haemorrhage	AmBisome	Died
						Itraconazole	
						AVR	
4^(5)^	2006	75 male	PPM	PPM	Pulmonary emboli	Voriconazole	Survived
						PPM removal	
5^(9)^	2010	50 male	Rheumatic disease	Mitral	Septic shock	AmBisome	Died
						MVR	
6^(5)^	2010	29 female	ALL	Mitral	Endophthalmitis, osteomyelitis & cerebral emboli	AmBisome	Died
						Voriconazole	
						MVR	
7^(10)^	2013	35 male	Renal transplant	Aortic	Meningitis	AmBisome	Died
						Voriconazole	
8^(8)^	2014	66 female	AML	Mitral	Sinusitis, pulmonary & splenic emboli	Voriconazole	Died
						Terbinafine	
Case	2014	75 female	Ovarian cancer	Aortic	Cerebral emboli	Voriconazole	Died

In this patient, the diagnosis of infective endocarditis was based on the Duke clinical criteria, as no pathological specimens were available. She fulfilled the criteria as follows: 1 major, in the form of echocardiographic findings on TOE, along with 3 minor; fever, embolic phenomenon with cerebral lesions, and positive blood cultures for *Lomentospora prolificans.* Despite advanced malignancy, marantic (non-bacterial thrombotic endocarditis) was considered very unlikely due to the negative FDG-PET imaging for active malignancy.

Matrix-assisted laser desorption ionisation-time of flight mass spectromectry (MALDI-TOF) is being increasingly utilised as an important and accurate identification technique for *Scedosporium* species, which may assist with optimising early empiric antifungal treatment. In a recent study, MALDI-TOF (using the Andromas system) was able to identify 64 *Pseudallescheria* and *Scedosporium* isolates with 100 % accuracy [11]. Polymerase chain reaction-based restriction fragment methods have also proven useful in accurately identifying *L.prolificans,* particularly from small fungal concentrations [12].

There are no evidence based guidelines for treatment. Surgical excision is paramount to patient survival. In the setting of endocarditis, cardiothoracic intervention is required to excise the infected valve or cardiac device. *L.prolificans* is resistant to many classes of antifungals. Of the triazole agents currently available, including isavuconazole, voriconazole demonstrates the most effect against *L. prolificans* in-vitro*,* however the MIC_90_ remains >16 in most data sets [13, 14]. Despite in-vitro susceptibility, clinical efficacy is often poor [4]. This is in contrast to *S. aurantiacum* which displays considerably lower MIC_90_ values to voriconazole [14]*.* Combination therapy has been used with amphotericin and pentamidine, and voriconazole combined with terbinafine. Optimal length of treatment is unclear [1, 6, 13].

## Conclusion

Endocarditis caused by *Lomentospora prolificans* is a rare but emerging condition, with a high mortality and limited treatment options. Survival is documented in patients who have undergone aggressive surgical excision with valve replacement and anti-fungal therapy. Awareness of the increasing incidence of invasive *Lomentospora prolificans* infection, particularly in immunosuppressed patients, with a propensity to present late in infection and potential for endocarditis may prompt earlier echocardiography or FDG-PET imaging and guide empiric antifungal therapy.

## Consent

Written informed consent was obtained from the patient’s next of kin, her daughter, for publication of this report and accompanying images. A copy of the written consent is available for review by the Editor of this journal.

## Additional file

Additional file 1:
**Transoesophageal echocardiogram axial view demonstrating highly mobile, large pedunculated mass within the ascending aorta arising from the commissure of the right and non-coronary cusp of the aortic valve with a calcified base.** (AVI 37971 kb)

## Abbreviations

FDG-PET: Fluoro-D-glucose positron emission tomography
MALDI-TOF: matrix-assisted laser desorption ionisation-time of flight mass spectromectry
MIC: minimum inhibitory concentration
TOE: transoesophageal echocardiogram
TTE: transthoracic echocardiogram
